# Headache characteristics among patients with epilepsy and the association with temporal encephaloceles

**DOI:** 10.1016/j.ibneur.2022.11.003

**Published:** 2022-11-11

**Authors:** Patricia Graese, Milad Yazdani, Zeke Campbell

**Affiliations:** a Department of Neurology, Thomas Jefferson University, Philadelphia, PA, USA; b Department of Radiology, Medical University of South Carolina, Charleston, SC, USA; c Department of Neurology, Medical University of South Carolina, Charleston, SC, USA

**Keywords:** Encephalocele, Idiopathic intracranial hypertension, Epilepsy, Headache

## Abstract

**Purpose:**

Our aim was to determine if headaches characteristic of possible Idiopathic Intracranial Hypertension (IIH) and in general were more prevalent in patients with versus without temporal encephaloceles (TEs) among patients with epilepsy.

**Methods:**

Electronic medical records were reviewed retrospectively. Among 474 patients with epilepsy, 103 patients (21.7%) had at least one TE diagnosed on initial MRI or on retrospective review by a board-certified neuroradiologist, while 371 patients had no TE present. The patients were grouped into one of four categories depending on their headache characteristics (IIH-like, peri-ictal, other, or no headaches). Analysis of the categories was performed using a Chi Square test.

**Results:**

Patients with TEs were more likely to experience headaches of any type than no headaches and more likely to experience IIH-like headaches than to have other headaches or no headaches compared to patients without TEs. Interestingly, patients with TEs were also more likely to experience peri-ictal headaches compared to patients without TEs. However, patients with TEs were no more likely or unlikely to have other (non-IIH or peri-ictal) headaches vs no reported headaches and were not more or less likely to have elevated opening pressure.

**Conclusion:**

On retrospective review, patients with TEs and epilepsy were more likely to exhibit headache features characteristic of IIH or to have peri-ictal headaches compared to epilepsy patients without TEs. These findings underscore the need for careful and thorough history of associated headaches in patients with epilepsy so that those patients at risk for TEs can undergo careful inspection of MRI to evaluate for their presence, which may represent a focus for seizures.

## 1 Introduction

Encephaloceles are herniations of brain matter and associated dura through a skull defect. Temporal encephaloceles (TEs), located within the middle cranial fossa correlating with the temporal brain parenchyma, have been implicated as a potential seizure focus in refractory temporal lobe epilepsy (RTLE) (Campbell et al., 2018, Panov et al., 2016, Toledano et al., 2016). Although consensus on the prevalence of spontaneous encephaloceles has varied, it has recently been reported in up to 10–23% of RTLE patients (Toledano et al., 2016, Saavalainen et al., 2015). In many cases, these encephaloceles were only identified once magnetic resonance imaging (MRI), often with epilepsy-specific protocol including thin cut coronal images through the temporal lobes, was reviewed carefully and sometimes retrospectively by experienced neuroradiologists (Campbell et al., 2018, Toledano et al., 2016).

Idiopathic intracranial hypertension (IIH) has been proposed as a potential pathway in the formation of spontaneous encephaloceles (Brainard et al., 2012, Schlosser et al., 2006, Maralani et al., 2012, Schlosser and Bolger, 2003, Bialer et al., 2014). Our aim was to determine if patients with focal epilepsy and TEs were more likely to exhibit particular headache characteristics compared to patients without TEs, as patients with IIH often display signs or symptoms suggestive of increased intracranial pressure (Thurtell, 2017).

## 2 Materials and methods

### 2.1 Patients

Electronic medical records were reviewed on a total of 474 patients. Patients were included in the study if they had been presented at a multidisciplinary refractory epilepsy conference between 2010 and 2016 (423 patients), while the rest were evaluated in a comprehensive epilepsy clinic and included if they had at least one temporal encephalocele identified retrospectively on MRI. All patients had either ictal scalp electroencephalogram (EEG), intracranial EEG, or both performed to confirm the diagnosis of epilepsy. A total of one hundred three patients had at least one TE noted on MRI brain confirmed by a board-certified neuroradiologist. No patients meeting the above criteria were excluded.

### 2.2 MRI imaging protocol

**Fig. 1:**
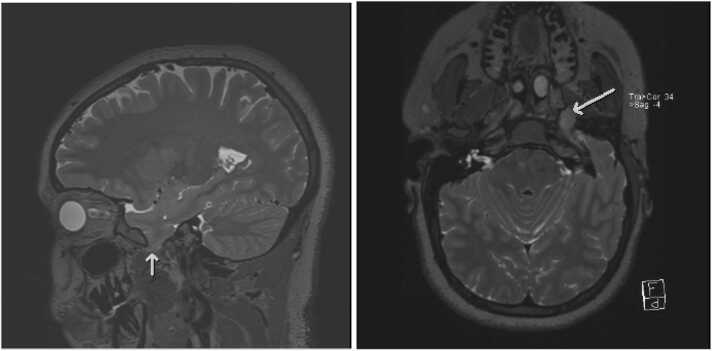
Axial and sagittal T2 SPACE images show encephalocele in left middle cranial fossa (white arrows).

The imaging data was gathered in a prior study completed at the same academic center (Campbell et al., 2018). All patients underwent MRI imaging according to an epilepsy specific protocol. They were scanned on either a 1.5 T Avanto or Aera or 3 T Skyra or Verio (Siemens, Erlangen, Germany) MRI scanner. In addition to the routine sequences, our epilepsy specific protocol includes sagittal T2 FLAIR (TI, 1800 ms; TE, 387 ms; TR, 5000 ms) and sagittal T2 (TE, 408 ms; TR, 3200 ms) sampling perfected with application optimized contrasts by using different flip angle evolution (SPACE; Siemens) sequences of the whole brain with section thickness of 0.9 mm and voxel size of 0.9 × 0.9 × 0.9 mm, which were subsequently reconstructed in axial and coronal planes. These additional sequences were helpful in identifying small encephaloceles. Fig. 1.

### 2.3 Headache categorization

Historical information was reviewed; patients were grouped into one of four categories depending on their headache characteristics. Patients with IIH-like headaches were defined by having an elevated opening pressure (OP) > 25 cm H_2_O OR two or more of the following associated descriptors: positional (worse on lying or improved on standing), worse in the morning, pulsatile tinnitus, or blurry vision. Patients with peri-ictal headaches included those that were restricted to the prodromal, ictal, or postictal phases. Patients with migraine, tension, medication-related or any other headaches with nondescript features fell within the other headache category. The last category consisted of patients with no headaches.

### 2.4 Statistical analysis

The four categories of headaches in patients with and without TEs were evaluated using a Chi Square test, as were the number of elevated OPs and non-elevated OPs in patients with and without TEs. Statistical significance was determined at P = 0.05.

## 3 Results

Among 474 patients with epilepsy, 103 (21.7%) patients were identified as having a TE. The comparison among headache types for patients with versus without TEs is shown in Table 1. Patients with TEs were more likely to experience headaches of any type than no headaches (p = 0.005) and more likely to experience IIH-like headaches than to have other headaches or no headaches compared to patients without TEs (P < 0.001). Interestingly, patients with TEs were also more likely to experience peri-ictal headaches compared to patients without TEs (p = 0.001). However, patients with TEs were no more likely or unlikely to have other headaches or no reported headaches (p = 1). A total of 24 patients underwent lumbar puncture with OP, in most cases for evaluation of headache, although in some cases the documented reason was not clear; no difference was observed in elevated or normal OP in patients with or without TEs (p = 0.057).

**Table 1:** Comparison of headache types in patients with versus without temporal encephaloceles.

	Total N = 474	TE N = 103	Without TE N = 371	p
Demographics				
Age (years)	36.7 ± 13.5	44.2 ± 13.7	36.7 ± 13.5	< 0.001
Male	189 (40.0%)	23 (19.4%)	166 (44.9%)	< 0.001
Headache types				
All headaches IIH-like	304 23	78 (75.7%) 17 (16.5%)	226 (60.9%) 6 (1.6%)	0.005 < 0.001
Peri-ictal	114	37 (35.9%)	77 (20.8%)	0.001
Other	167	24 (23.3%)	143 (38.6%)	0.004
None	170	25 (24.2%)	145 (39.2%)	0.005
Opening pressure				
Normal	10	5	5	
Elevated	14	12	2	0.057

## 4 Discussion

TEs and their relationship to epilepsy have become a rapidly increasing area of interest and potentially curable target for RTLE (Panov et al., 2016, Ranjan et al., 2019). The identification of clinical characteristics found in patients with TEs could prove useful in earlier detection and treatment, as well as contribute to our understanding of their pathogenesis. While certain demographic characteristics (elevated body mass index (BMI), female preponderance, older age and longer duration of epilepsy) and semiological features suggestive of mesiotemporal seizure onset are known to be associated with TEs, other clinical signs or symptoms specific for their presence had not been identified (Campbell et al., 2018, Panov et al., 2016, Paule et al., 2019).

In the absence of any neoplastic, inflammatory, traumatic or iatrogenic causes, IIH has been a favored explanation for spontaneous encephaloceles. Various factors have been studied to confirm this relationship. Patients with cerebrospinal fluid (CSF) otorrhea or other spontaneous CSF leaks from increased intracranial pressure have had higher incidences of encephaloceles (Brainard et al., 2012, Schlosser et al., 2006), and higher BMI has been noted in patients with TEs, also an associated factor in IIH (Campbell et al., 2018, Schlosser and Bolger, 2003, Scurry et al., 2007). Radiographic findings included in the modified Dandy Criteria for IIH are an empty sella turcica, optic nerve sheath with filled out CSF spaces, and smooth-walled non flow-related venous sinus stenosis or collapse (Maralani et al., 2012). Studies have shown that encephaloceles may be more commonly found in the context of these other findings than previously thought (Campbell et al., 2018, Schlosser and Bolger, 2003). Therefore, TEs in the context of no other clear explanation of increased intracranial pressure could be suggestive of IIH or vice versa. A recent study that appears to run contrary to our findings revealed the presence of radiological features of IIH without associated clinical manifestations, for which it is hypothesized that evacuation of CSF through the bone defect relieves the elevated ICP thereby averting the development of clinical manifestations (Martinez-Poles et al., 2021). While it is possible that some CSF could be leaked through a bony defect, it is unclear how patients would develop the noted radiological signs of IIH without clinical phenomena in the context of normal ICP. Specific for patients with RTLE, inquiring about symptoms of IIH including positional headaches (worse with lying, improved with standing), headaches worse in morning, pulsatile tinnitus, and change in vision may be useful. If such symptoms are present, a more deliberate search for TEs on MRI may be of higher yield, particularly since it has been found in a study by Campbell et al. that only 7 of 418 patients with refractory epilepsy had TEs noted on initial imaging, while 52 had TEs when MRI was reviewed by a board-certified neuroradiologist with the explicit intent of looking for TEs (Campbell et al., 2018). Additionally, it was shown that detection of TEs was increased with 3 T compared to 1.5 T MRI.

Interestingly, patients with TEs were also more likely to have peri-ictal headaches despite an overall similar prevalence of nondescript or absence of headache. Peri-ictal headaches are a long described phenomenon but only recently have been officially added to the International Classification of Headache Disorders (Parisi et al., 2019). The ictal state in patients with focal seizures is associated with increased cerebral metabolism and a corresponding increase in cerebral perfusion by 300% (Parisi et al., 2019, Hajek et al., 1991), which could theoretically result in increased intracranial pressure in the peri-ictal state. Additionally, critically ill patients who are monitored have intracranial pressures that often spike during seizures (Gabor et al., 1984). For patients with minimally elevated or borderline opening pressures who might not fulfill clinical criteria for IIH, this temporary spike in pressure could be enough to create pain and lead to headaches. Specific interrogation to the characteristics of ictal headaches may shed light on the intracranial pressure status of epilepsy patients with and without TEs.

Ultimately, an elevation in OP is the definitive factor needed to diagnosis IIH. While different parameters are sometimes given to patients with a BMI > 30 for elevated pressure, OP greater than 25 cm H_2_0 is generally accepted as elevated (Thurtell, 2017, Corbett and Mehta, 1983). Although no statistical difference was achieved in comparing patients with elevated versus normal OP in those with or without TEs, there was a trend toward patients with TEs having elevated OP (70.5%) compared to those without TEs (28.5%). Due to the small subset of patients (24) who had a recorded OP, larger studies would be required to determine if this trend holds true. This study was also limited by its retrospective nature. The epilepsy patients were not all evaluated clinically by the same neurologist and recording of headache symptoms were not performed in a systematic fashion. Future prospective studies should include a standardized questionnaire to create uniformity.

## 5 Conclusions

On retrospective review, patients with TEs and epilepsy were more likely to exhibit headache features characteristic of IIH or to have peri-ictal headaches compared to patients without TEs. These findings underscore the need for careful and thorough history of associated headaches in patients with epilepsy so that those patients at risk for TEs can undergo careful inspection of MRI to evaluate for their presence, which may represent a focus for seizures. While our study provides information on the clinical presentation of patients with epilepsy and TEs, further studies aimed at determining the epileptogenicity and appropriate management of TEs are needed.

## CRediT authorship contribution statement

**Patricia Graese**: Methodology, Investigation, Analysis, Writing − original draft. **Milad Yazdani**: Investigation, Writing − review & editing. **Zeke Campbell**: Conceptualization, Methodology, Analysis, Writing − review & editing.

## Declarations of interest

None.
